# Cellular and Molecular Mechanisms of Diabetic Atherosclerosis: Herbal Medicines as a Potential Therapeutic Approach

**DOI:** 10.1155/2017/9080869

**Published:** 2017-08-13

**Authors:** Jinfan Tian, Yanfei Liu, Yue Liu, Keji Chen, Shuzheng Lyu

**Affiliations:** ^1^Department of Cardiology, Beijing Anzhen Hospital, Capital Medical University, Beijing 100029, China; ^2^Graduate School, Beijing University of Chinese Medicine, Beijing 100029, China; ^3^Cardiovascular Disease Centre, Xiyuan Hospital, China Academy of Chinese Medical Sciences, Beijing 100091, China

## Abstract

An increasing number of patients diagnosed with diabetes mellitus eventually develop severe coronary atherosclerosis disease. Both type 1 and type 2 diabetes mellitus increase the risk of cardiovascular disease associated with atherosclerosis. The cellular and molecular mechanisms affecting the incidence of diabetic atherosclerosis are still unclear, as are appropriate strategies for the prevention and treatment of diabetic atherosclerosis. In this review, we discuss progress in the study of herbs as potential therapeutic agents for diabetic atherosclerosis.

## 1. Introduction

Cardiovascular diseases (CVDs), including atherosclerosis, are important complications of diabetes and the leading causes of mortality in patients with diabetes. Systemic factors accompanying diabetes, such as dyslipidaemia and hypertension, are thought to affect the development of diabetic vascular diseases. In addition, insulin resistance and excess production of advanced glycosylation end products (AGEs) contribute to disorders of lipid metabolism, oxidative stress, endothelial dysfunction, monocyte recruitment, foam cell formation, phenotype changes in vascular smooth muscle cells (VSMCs), and thrombosis formation [1]. In addition, diabetes and atherosclerosis exhibit common pathologies, although the underlying mechanisms are still being explored.

Botanical and natural drugs have a long, documented history in treating diabetes and vascular diseases. The vascular-protective properties of herb medicines include their ability to scavenge free radicals, inhibit apoptosis, and reduce inflammation and platelet aggregation [2]. Most recently, Li et al. [3] uncovered the antidiabetes effect of artemisinins, and the mechanism involves driving the in vivo conversion of pancreatic cells into functional *β*-like cells by enhancing GABA signalling. Furthermore, owing to the multitarget effects and comprehensive sources of herbal medicines, it remains of utmost importance to improve our understanding of their potential use in the treatment of diabetes and diabetic atherosclerosis despite their reported side effects. Since diabetic atherosclerosis is a multifactorial disease, in this review, we first discuss the cellular and molecular mechanisms for the pathogenesis of diabetic atherosclerosis and then the progress in the study of herbs as potential therapeutic agents for diabetic atherosclerosis.

## 2. Cell Types Involved in Diabetic Atherosclerosis

### 2.1. Myeloid Cells

Diabetes and atherosclerosis are chronic inflammatory conditions. Myeloid cells (neutrophils, monocytes, and macrophages) are involved in both atherosclerosis and diabetes. The migration of circulating monocytes into the vessel wall is critical for the development of diabetic atherosclerosis. Moreover, intercellular cell adhesion molecule-1(ICAM-1), chemoattractant protein-1(MCP-1), and macrophage migration inhibitory factor (MIF), which regulate the adhesion of monocytes, are dysregulated in hyperglycemia-induced atherosclerosis in animal models. Increased foam cells derived from macrophages promote the acceleration of atherosclerotic lesions in diabetic ApoE^−/−^ mice [4, 5]. A more inflammatory monocyte/macrophage phenotype with secretion of higher levels of proinflammatory cytokines was detected in both animal models and patients with diabetes mellitus [6]. Increases in long-chain acyl-COA synthetase 1 (ACSL1), toll-like receptor (TLR) 2, and TLR4 contribute to the increased inflammatory monocyte/macrophage phenotype in the context of diabetes [6]. Neutrophil infiltration also has a role in diabetic atherosclerosis. In addition, T-cell function is closely related to atherosclerosis in the diabetic environment, and inflammatory monocytes have been shown to activate Th17 cells under diabetic conditions [7].

### 2.2. Endothelial Cells

Endothelial dysfunction due to inflammation and oxidative stress is a crucial characteristic in diabetes mellitus-linked atherosclerosis. Endothelial dysfunction is associated with decreased nitric oxide (NO) availability, either through loss of NO production or NO biological activity [8, 9]. The excess generation of free oxygen radicals leads to apoptosis in endothelial cells [9]. In hyperglycemia, chronic inflammation increases vascular permeability, promotes the generation of adhesion molecules and chemokines, and stimulates accumulation of monocytes in the artery wall. The interleukin-1 (IL-1) antagonist anakinra improves endothelial dysfunction in diabetic animals via attenuation of the proinflammatory enzymes cyclooxygenase(COX) and inducible nitric oxide synthase (iNOS) triggered by diabetes in the vascular wall [10, 11].

### 2.3. Smooth Muscle Cells

Proliferation and accumulation of smooth muscle cells are detected in both type 1 and type 2 diabetes mellitus. However, it is still unclear whether changes in smooth muscle cells are a result of the diabetic environment directly or are caused by endothelial injury and macrophage recruitment. According to a report by Chen et al. [12], various concentrations of glucose (5.6, 11.1, 16.7, and 22.2 mM) increase the proliferation of vascular smooth muscle cells (VSMCs) in a concentration-dependent manner after 48 h of incubation. Another study showed that after initial injury, growth factors and cytokines released by endothelial cells, inflammatory cells, and platelets promote changes in VSMC phenotypes, thereby enhancing VSMC proliferation and migration [13]. Additionally, aortic smooth muscle cells isolated from NOX^−/−^ApoE^−^ mice exhibit a dedifferentiated phenotype, including loss of contractile gene expression [14].

### 2.4. Platelets

Accumulating evidence has shown that platelet hyperreactivity is a crucial cause of diabetic atherosclerosis in both animal models and diabetic patients. Enhanced platelet aggregation and synthesis of thromboxane A2 were detected within days of streptozotocin- (STZ-) dependent induction of diabetes in a rat model [15]. Platelets from patients with diabetes have been shown to have decreased sensitivity to antiaggregation agents, such as prostacyclin (PGI2) and NO [16]. Glycated low-density lipoprotein- (GlyLDL-) and hyperinsulinemia-induced impairment of calcium homeostasis, activation of protein kinase C (PKC), increased generation of reactive oxygen species (ROS), and decreased NO bioactivity result in hyperactivation of platelets [17]. According to a report by Wang et al., a significant correlation between plasma CTRP9 concentrations (a novel adiponectin paralog) and platelet aggregation amplitude was observed in high-fat diet-induced diabetic C57BL/6J mice. Enhancing CTRP9 production and/or exogenous supplementation of CTRP9 may protect against diabetic cardiovascular injury via inhibition of abnormal platelet activity [18].

## 3. Molecules or Signal Transduction Pathways

### 3.1. Molecules

#### 3.1.1. AGEs

The detrimental effects of hyperglycemia can be attributed to the biochemical consequences of intracellular metabolism associated with excess glucose, including nonenzymatic glycation with formation of AGEs [1]. AGEs are heterogeneous compounds that interact with the arterial wall through receptors for AGE (RAGEs). Formation of GlyLDLs and other AGEs induces uptake of proatherogenic lipids by cultured aortic SMCs [19] and stimulation of RAGE and scavenger-receptor expression in macrophages [20]. Schmidt et al. found that the interactions between AGEs and RAGEs enhance the adhesion of monocytes to endothelial cells via stimulating the expression of nuclear factor-*κ*B (NF-*κ*B-) dependent proinflammatory and prothrombotic molecules [21]. Consequently, the AGE/RAGE axis contributes to diabetic atherosclerosis by attracting monocytes to the vascular intima, increasing oxidative stress, inducing endothelial dysfunction, and promoting vascular wall remodeling [22, 23]. Menini et al. have shown that d-carnosine-octylester- (DCO-) attenuated AGE formation is related to its reactive carbonyl species- (RCS-) quenching activity [24]. Moreover, they also revealed that DCO treatment attenuated lesion size, necrotic area, and apoptotic cells in diabetic ApoE-null mice. These protection effects were more effectively achieved by early treatment (60 mg/kg body weight, from weeks 1 to 11, DCO early) than by late treatment (60 mg/kg body weight, from weeks 9 to 19, DCO late) [25]. Zhu et al. [26] demonstrated that immunized diabetic ApoE^−/−^ and low-density lipoprotein (LDL) receptor knockout (LDLR)^−/−^ mice with AGE-LDL significantly reduced atherosclerosis, indicating that vaccination with AGE-LDL may offer a novel approach for the treatment of atherosclerosis in patients with diabetes. Inhibition of RAGE using murine-soluble RAGE (sRAGE) attenuates atherosclerotic lesions in STZ-induced diabetic ApoE^−/−^ mice and ApoE^−/−^/db/db mice [27]. These findings further supported the roles of AGE and RAGE in the macrovascular complications of diabetes, and blockade of RAGE may be a potential therapeutic strategy in diabetic atherosclerosis.

#### 3.1.2. ACSL1

A recent study showed that monocytes and macrophages expressed increased levels of ACSL1 (an enzyme that catalyzes the thioesterification of fatty acids) in both diabetic mouse models and human subjects [6]. ACSL1 is markedly induced by the TLR4 ligand lipopolysaccharide (LPS) in isolated macrophages, suggesting that ACSL1 may be a downstream effector of TLR4 cascade in macrophages [28]. Myeloid-specific ACSL1 deficiency results in a specific reduction in 20:4-COA levels and completely prevents the increased release of prostaglandin E2 (PGE2) and increased inflammatory phenotype in monocytes and macrophages from diabetic mice, suggesting that the inflammatory phenotype is associated with increased expression of ACSL1. In addition, increased chemokine (C-C motif) ligand 2 (CCL2) secretion from macrophages in diabetic mice is completely prevented by ACSL1 deficiency, supporting that monocyte recruitment is reduced by ACSL1 deficiency [6]. Kanter et al. [29] demonstrated that ACSL1 could directly influence ATP-binding cassette transporter A1 (ABCA1) levels and cholesterol efflux in mouse macrophages. Mouse macrophages deficient in ACSL1 displayed increased ABCA1 levels and increased apolipoprotein A-I-dependent cholesterol efflux in the presence of unsaturated fatty acids compared with those of wild-type mouse macrophages. Conversely, overexpression of ACS1 led to reduced ABCA1 levels and reduced cholesterol efflux in the presence of unsaturated fatty acids. Taken together, the reduced levels of cholesterol efflux and expression of ABCA1 in mouse macrophages in the context of diabetes and elevated fatty load were partly mediated by ACSL1.

#### 3.1.3. Paraoxonase (PON1)

In the state of high oxidative stress, such as STZ-induced diabetes, serum PON1 and arylesterase activities were reduced [30, 31]. Decreased serum PON activity is related to glycation and glycol oxidation of high-density lipoprotein (HDL) in the hyperglycemic state, thus leading to impairment of HDL activity, such as protection of LDL from oxidation, cholesterol efflux from cells, and inhibition of monocyte migration to endothelial cells [32, 33]. Taş et al. have demonstrated that vitamin B6 supplementation enhances serum PON1 and arylesterase activities, which could be related to the potential direct effects of this vitamin on the enzyme and/or to its ability to reduce oxidative stress [34]. These results suggested that protection of PON1 from inactivation may be a potential therapeutic approach for the treatment of diabetic atherosclerosis.

#### 3.1.4. Insulinotropic Polypeptide (GIP)

As illustrated previously, high glucose accelerates atherosclerosis and foam cell formation. GIP potently stimulates insulin release from the pancreas under conditions of normal glucose tolerance. However, under diabetic conditions, the activity of GIP is reduced [35]. Thus, GIP is thought to be involved in diabetic atherosclerosis. According to Nogi et al. [36], chronic administration of GIP in vivo attenuates macrophage-driven atherosclerotic lesions in STZ-induced diabetic ApoE^−/−^ mice, although this effect is abolished by cofusion with a GIPR antagonist, [Pro^3^]GIP. GIP infusion attenuates foam cell formation in both diabetic ApoE^−/−^ mice and db/db mice. In vitro treatment with GIP (1 nM) reduces foam cell formation by 15% in macrophages from diabetic ApoE^−/−^ mice.

#### 3.1.5. MicroRNA (miRNA)

miRNA is a class of conserved 19–25-nucleotide noncoding RNAs that regulate gene expression posttranscriptionally. Recently, researchers have demonstrated that miRNAs play important roles in diabetes and related complications (Table 1). According to a study by Distel et al., treatment of STZ-induced diabetic LDLR^−/−^ mice with anti-*miR-33* decreases plaque macrophage content and inflammatory gene expression in diabetic LDLR^−/−^ mice, accompanied by upregulation of ABCA1, which mediates cholesterol efflux from macrophages in the plaque [37]. Villeneuve et al. [38] first demonstrated the role of *miR-125b* in vascular complications of diabetes. They verified that *miR-125b*, which targets SUV39H1, led to increased levels of inflammatory genes, such as *IL-6* and MCP-1. *miR-125b* mimic significantly increased monocyte binding to smooth muscle cells in db/db mice. According to a study by Reddy et al. [39], the expression levels of *miR-200b* and *miR-200c* were increased, whereas Zeb1 protein levels were decreased in VSMCs and aortas from db/db mice relative to those in control db/+mice. Transfection with *miR-200* mimic downregulated Zeb1, upregulated the inflammatory genes *COX-2* and *MCP-1*, and promoted monocyte binding in db/+ VSMCs. Both miR mimics and Zeb1 siRNA increased the proinflammatory response in db/db VSMCs. In contrast, *miR-200* inhibitors reversed the enhanced monocyte binding of db/db VSMCs. Moreover, *miR-504* significantly upregulated in db/db VSMCs compared with that in db/+VSMCs [40]. *miR-504* may enhance extracellular regulated protein kinase 1/2 (ERK1/2) activation by targeting Grb10 and thereby contribute to changes in the VSMC phenotype. According to Xu et al. [41], higher *miR-138* levels and reduced expression of silent information regulator 1 (SIRT1) were observed in SMCs isolated from db/db mice. Additionally, *miR-138* promotes smooth muscle cell proliferation and migration in db/db mice through downregulation of SIRT1, whereas transfection with *miR-138* inhibitor reverses these effects.

#### 3.1.6. Tribbles Homolog 3 (TRIB3)

The expression of TRIB3, a protein made up of 358 amino acids, is increased in patients and animals with type 2 diabetes [42]. Endoplasmic reticulum stress, an important feature of diabetes, has also been shown to increase TRIB3 expression, thus promoting cell death in response to endoplasmic reticulum stress [43].TRIB3 impairs insulin metabolic signalling by increasing serine phosphorylation of insulin receptor substrate 1 (IRS-1), reducing activation of phosphatidylinositol 3-kinase (PI3K)/Akt [44], or directly inhibiting the phosphorylation of Akt [45, 46].

**Figure 1:**
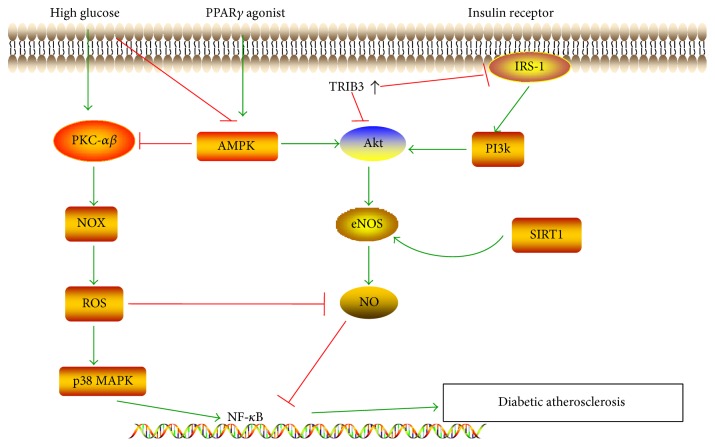
The NF-*κ*B signalling pathway is involved in the diabetic atherosclerosis. Decreased NO produced by eNOS is associated with diabetic atherosclerosis. PI3K and AMPK are upstream of Akt and eNOS. Under hyperglycemic conditions, PKC activates NOX, the major source of reactive ROS production, leading to the activation of p38 MAPK and NF-*κ*B and decrease in NO bioavailability. AMPK suppression in the context of high glucose contributes to the downregulation of the Akt/NO cascade. Increased levels of TRIB3 inhibit the phosphorylation of Akt directly or by reducing activation of PI3K/Akt. SIRT1 stimulates NO production by deacetylating eNOS at lysine residues or by upregulating AMPK. PPAR*γ* agonists activate AMPK, which in turn increases the bioactivity of eNOS and prevents PKC-activated NOX caused by high glucose. PKC: protein kinase; NOX: NADPH oxidase; TRIB3: Tribbles homolog 3; ROS: reactive oxygen species; p38 MAPK: p38 mitogen-activated protein kinase; PI3K: phosphatidylinositol 3-kinase; AMPK: AMP-activated protein kinase; eNOS: endothelial NO synthase; IRS-1: insulin receptor substrate 1; SIRT1: silent information regulator 1; PPAR*γ*: peroxisome proliferator-activated receptor *γ.*

Several mechanisms are involved in TRIB3-dependent promotion of atherosclerotic lesions. TRIB3 impairs IRS-1/Akt signalling in endothelial cells mediated by insulin [47], leading to reduced endothelial nitric oxide synthase (eNOS) and NO bioavailability [46], which is associated with endothelial dysfunction and increased leukocyte adhesion to endothelial cells, important steps for atherosclerotic lesion formation (Figure 1) [9]. In addition, TRIB3 is involved in lipid metabolism and macrophage apoptosis, an important feature for vulnerable plaques [48, 49]. According to a study by Wang et al. [49], silencing of TRIB3 in STZ plus diet-induced diabetic ApoE^−/−^/LDLR^−/−^mice significantly decreases insulin resistance and blood glucose and reduces the numbers of apoptotic cells and macrophages in atherosclerotic lesions. Consequently, silencing of TRIB3 attenuates the atherosclerosis burden and promotes plaque stability in diabetic mice. Thus, TRIB3 is a promising target for the treatment of diabetic atherosclerosis.

### 3.2. Signalling Pathways

#### 3.2.1. The Janus Kinase (JAK)/Signal Transducers and Activators of Transcription (STAT) Cascade

JAK/STAT is an essential intracellular pathway that regulates leukocyte recruitment, foam cell formation, and proliferation and migration of VSMCs, which are important features in atherosclerosis [50–52]. STAT isoforms have been found in the atherosclerotic lesions in both humans and animal models [53, 54]. STAT signalling cascade contributes to the macrophage apoptosis in advanced atherosclerotic plaque [55]. Inhibition of JAK2, STAT1, and STAT3 reduces lesion size and neointimal hyperplasia [56, 57]. Moreover, JAK/STAT is also a pivotal inflammatory mechanism through which hyperglycemia contributes to the pathogenesis of diabetes mellitus and its vascular complications [58–60]. High glucose stimulates endothelial IL-6 secretion via redox-dependent mechanisms, which may consequently induce STAT3 activation and ICAM-1 expression; the specific STAT3 inhibitor SI-201 (20 *μ*M) suppresses high glucose-induced ICAM expression in cultured human umbilical vein endothelial cells (HUVECs) [61]. The suppressor of cytokine signalling (SOCS) family regulates JAK/STAT signalling through STAT binding, kinase inhibition, targeting for proteasomal degradation, or direct suppression of JAK tyrosine kinase activity [62, 63]. According to a report by Recio et al. [64], SOCS1 peptide inhibits STAT1/STAT3 activation and target gene expression in VSMCs and macrophages and blocks the migration and adhesion of macrophages in vitro. Their results showed that intraperitoneal injection of SOCS1 into STZ-induced diabetic ApoE^−/−^ mice (ages 8 and 22 weeks) for 6–10 weeks suppressed STAT1/STAT3 activation in atherosclerotic plaques and significantly attenuated lesion size for both early and advanced lesions. The accumulation of lipids, macrophages, and T lymphocytes was decreased following treatment with SOCS1 peptide, whereas collagen and smooth muscle cell content were significantly increased. Thus, the SOCS/JAK/STAT cascade was a key molecular mechanism through which diabetes promoted atherosclerotic plaque formation, and SOCS1 endogenous protein may be a feasible target for modulating inflammation-related complications of diabetes mellitus. Approaches to supplement SOC1 or mimic native SOCS1 function may have therapeutic effects on accelerated atherosclerosis in diabetes.

#### 3.2.2. The eNOS/NO Pathway

NO produced by eNOS is an important vasodilator that possesses multiple antiatherosclerotic properties. eNOS-derived NO has been shown to inhibit platelet aggregation, block vascular inflammation by inhibiting the activation of NF-*κ*B [65], and suppress VSMC proliferation. As illustrated previously, decreased bioactivity of NO is associated with exposure of endothelial cells to high-glucose concentration [9]. High-glucose levels block endothelial injury repair by circulating endothelial progenitor cells (EPCs) through decreasing eNOS/NO bioavailability [9, 66, 67]. According to a study by Sun et al. [68], the Akt kinase inhibitor (GSK690693) inhibited Akt and eNOS phosphorylation, suggesting that Akt may be necessary to activate eNOS. The PI3K inhibitor LY-29402 inhibits vaspin-induced eNOS and Akt phosphorylation, suggesting that PI3K acts upstream of Akt activation and eNOS. Additionally, vaspin induces endothelial protective effects via the PI3K/Akt/eNOS pathway. Ouchi et al. [69] showed that adiponectin-induced Akt phosphorylation, eNOS phosphorylation, and cell migration and differentiation in HUVECs were abolished when the cells were transduced with a dominant-negative form of AMP-activated protein kinase (AMPK). However, AMPK phosphorylation was not affected by dominant-negative transduction in HUVECs, suggesting that AMPK acts upstream of Akt in the Akt/eNOS/NO pathway to regulate endothelial function under conditions of hyperglycemia. SIRT1 is a class III histone deacetylase that has been shown to stimulate NO production by deacetylating eNOS at lysine residues [70] or by mediating the activation of AMPK [71]. Yang et al. [70] found that SIRT1 had a positive role in improving the expression of eNOS impaired by high glucose, and the low level of NO in endothelial cells cultured in the presence of high glucose may be partly related to the decreased expression of SIRT1 (Figure 1()).

#### 3.2.3. The Mitogen-Activated Protein Kinase (MAPK) Pathway

The MAPK pathway, including p38 MAPK, ERK, and c-Jun N-terminal kinase (JNK) branches, is involved in vascular inflammation. ERKs are typically initiated by Ras, which can be stimulated by inflammatory cytokines from high-glucose injured endothelial cells, leading to the proliferation of SMCs [72–75]. p38 MAPK activation is associated with diabetes and its complications, and the detrimental effects of high glucose can be blocked by coincubation with a p38 MAPK inhibitor [67]. Moreover, p38 MAPK has been shown to be involved in diabetic atherosclerosis. Hyperglycemic culture conditions accelerate the onset of EPC senescence, leading to impairment of endothelial repair, potentially through the activity of the p38 MAPK pathway [76]. Microparticles (MPs), which are submicron membrane vesicles (0.1–1 *μ*m) shed from the plasma membrane of activated or apoptotic cells, are significantly increased in the presence of high-glucose levels. According to a study by Jansen et al. [77], p38 MAPK is activated to phospho-p38MAPK within 30 min when human coronary endothelial cells (HCAECs) are treated with “injured” EMP (iEMP, MPs derived from glucose-treated HCAECs), whereas there is no change following treatment with normal endothelial cell-derived MPs (EMPs). iEMP-induced expression of ICAM-1 and VCAM-1 and monocyte adhesion to HCAECs were significantly reduced by pretreatment of HCAECs with the p38MAPK inhibitor SB-203580 (1 *μ*m). Consequently, these results showed that p38MAPK was involved in iEMP-induced endothelial dysfunction and monocyte adhesion. Further studies have shown that iEMP increases ROS production through NADPH oxidase (NOX) activation in endothelial cells, thus leading to activation of p38 MAPK. The p38 MAPK signalling pathway in diabetic atherosclerosis is illustrated in Figure 1.

#### 3.2.4. The Protein Kinase (PKC) Pathway

High concentrations of glucose and nonesterified fatty acids result in activation of PKC. Active PKC is involved in vascular inflammation through the generation of proinflammatory cytokines and chemokines. PKC activates NOX, the major source of ROS production in high-glucose stress, thus leading to the activation of signalling pathways such as ERK, p38 MAPK, and NF-*κ*B and decreased NO bioavailability (Figure 1) [78, 79]. ROS not only activate p38 MAPK but also act as an agonist to activate the nucleotide-binding domain-like receptor 3 (NLRP3) inflammasome, further disrupting endothelial function. These effects could be prevented by AMPK [80]. Durpès et al. [78] showed that PKC*β* decreases the expression of IL-18-binding protein (IL-18BP), a molecule involved in a negative feedback mechanism in response to elevated IL-18 production, thus enhancing the production of cytokines and cellular adhesion molecules, which promote atherosclerotic plaque formation and instability in STZ-induced diabetic ApoE^−/−^ mice. Kong et al. [81] found that activated plasma membrane-bound PKC*β* is elevated in the aortas of low-dose STZ-induced hyperglycemic ApoE^−/−^ mice and that pharmacological inhibition of PKC*β* attenuates atherosclerotic lesions in hyperglycemic ApoE^−/−^ mice. Deficiency of PKC*β* blocks the upregulation of Egr-1, ERK1/2, and JNK and results in diminished lesional macrophages and CD11c-expressing cells in diabetic ApoE^−/−^ mice. In vitro, inhibitors of PKC*β* and ERK1/2 significantly decrease high glucose-induced expression of CD11c, CCL2, and IL-1*β* in U937 macrophages. These studies suggest that selective PKC*β* inhibitors may have potential therapeutic effects in diabetes-associated atherosclerosis.

#### 3.2.5. The Peroxisome Proliferator-Activated Receptor (PPAR)*γ* Signalling Pathway

Accumulating evidence has shown that PPAR*γ* has protective effects in both diabetes and atherosclerosis. In a combined diabetes/atherosclerosis mouse model, PPAR*γ* agonists were found to exert antiatherogenic effects independent of a reduction in insulin resistance and plasma glucose [82], indicating that attenuation of insulin resistance is not the only mechanism through which PPAR*γ* functions as an antiatherognic agent. PPAR*γ* agonists activate AMPK, which in turn increases the bioactivity of eNOS and prevents PKC-activated NOX caused by high glucose [80, 83]. Pioglitazone downregulates RAGE expression and inhibits ROS production and NF-*κ*B activation via PPAR*γ* activation, which may prevent the inflammatory effects of the AGE/RAGE system in diabetes [84]. Recent studies have shown that pioglitazone attenuates platelet-derived growth factor (PDGF)-induced VSMC proliferation through AMPK-dependent and -independent inhibition of mammalian target of rapamycin (mTOR)/p70S6K and ERK signalling [85]. Furthermore, PPAR*γ* agonists have been reported to promote cholesterol efflux from macrophages via upregulation of ABCA1 expression [86, 87]. The PPAR*γ* signalling pathway in antiatherosclerosis under hyperglycemic conditions is illustrated in Figure 1.

#### 3.2.6. The Nuclear Factor of Activated T Cells (NFAT) Signalling Pathway

NFAT proteins are a family of Ca^2+^/calcineurin-dependent transcription factors first characterized in Tlymphocytes as inducers of cytokine gene expression. There are four well-characterized members of the NFAT family, which function in VSMC proliferation in the context of atherosclerosis and hypertension and have roles in glucose and insulin homeostasis [88]. According to a study by Nilsson et al., in intact cerebral arteries, raising the extracellular glucose concentration from 11.5 mM (control) to 20 mM [HG] for 30 min significantly increases NFAT nuclear accumulation, accompanied by enhanced transcriptional activity. UTP and UDP mediate glucose-induced NFAT activation via P2Y receptors. High-glucose concentrations downregulate glycogen synthase kinase 3 (GSK) *β* and JNK activity, leading to decreased export of NFATc3 from the nucleus and enhanced robust NFATc3 nuclear accumulation, representing another mechanism for glucose-induced NFAT activation [89]. NFATc3 is activated by hyperglycemia, thereby inducing the expression of osteopontin (OPN), a cytokine that promotes diabetic atherosclerosis [90]. Zetterqvist et al. demonstrated a link between NFAT activation and diabetic atherosclerosis using STZ-induced diabetic ApoE^−/−^ mice. In vivo treatment with the NFAT inhibitor A285222 (0.29 mg/kg/day i.p.) for 4 weeks prevented diabetes-associated atherosclerosis lesions in the aortic arch independent of blood glucose lowering, accompanied by decreased expression of IL-6, OPN, MCP-1, and ICAM-1 and the macrophage markers CD68 and tissue factor (TF) in the aortic arch. These findings revealed that the NFAT signalling pathway may be a promising target for the treatment of diabetes-associated atherosclerosis [91].

#### 3.2.7. The Nrf2 Signalling Pathway

**Figure 2:**
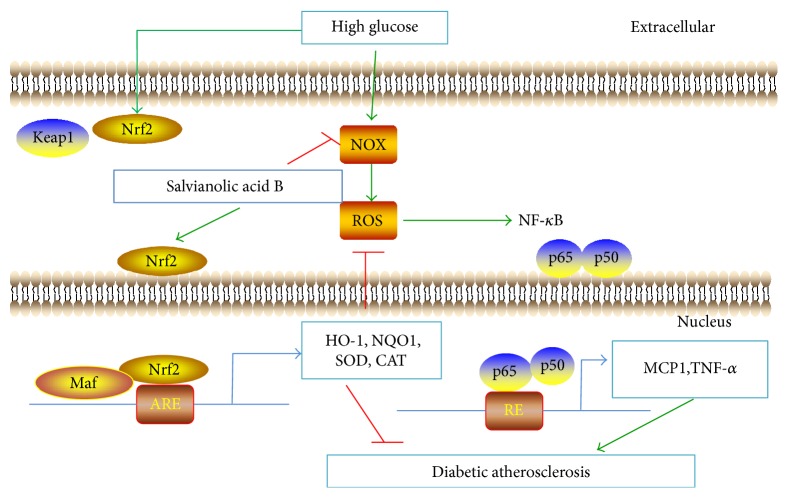
Salvianolic acid B protects against diabetic atherosclerosis via the Nrf2 signalling pathway. Under normal conditions, Nrf2 constitutively binds to keap1, a negative regulator in the cytosol. Under high-glucose stress, Nrf2 is released from keap1 and translocates to the nucleus, enhancing the transcriptional activity of NADPH: quinine oxidoreductase 1 (NQO1), heme oxygenase-1 (HO-1), superoxide dismutase (SOD), and catalase (CAT). These antioxidant enzymes decrease the levels of ROS. Salvianolic acid B is capable of upregulating Nrf2 activity or inhibiting NOX generation directly. ARE: antioxidant-responsive element; HO-1: heme oxygenase-1; NQO1: quinine oxidoreductase 1; SOD: superoxide dismutase; CAT: catalase.

Ungvari et al. demonstrated the vasoprotective role of Nrf2 in diabetes using Nrf2^−/−^ mice. They showed that the expression of Nrf2 downstream genes was significantly upregulated in diabetic Nrf2^+/+^ mice, but not in diabetic Nrf2^−/−^ mice [92]. Under normal conditions, Nrf2 constitutively interacts with keap1, a negative regulator, for ubiquitination and degradation in the cytosol. Under high-glucose stress, Nrf2 is released from keap1 and translocates to the nucleus and subsequently binds to antioxidant-responsive elements (ARE); this results in increased transcription of genes such as NADPH: quinine oxidoreductase 1 (NQO1), heme oxygenase-1 (HO-1), superoxide dismutase (SOD), and catalase (CAT). These antioxidant enzymes decrease the levels of ROS, thus attenuating diabetic atherosclerosis (Figure 2) [93]. These results suggest that Nrf2 activators may have efficacy in the management of diabetic atherosclerosis.

## 4. Herbal Medicines: Promising Therapeutic Agents for the Management of Diabetic Atherosclerosis

### 4.1. *Ginkgo biloba*

**Figure 3:**
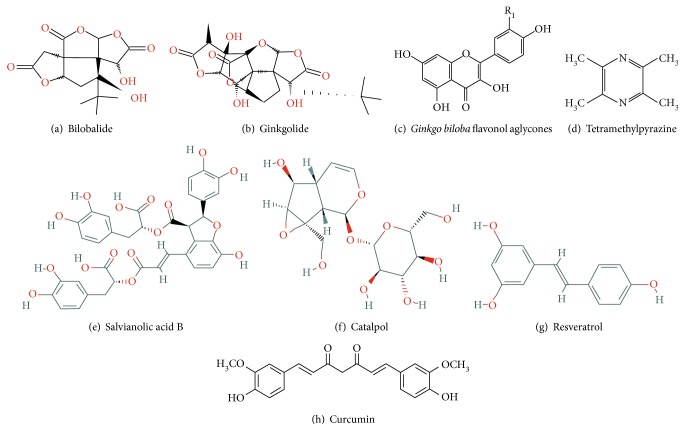
Molecular structure of the compounds described in this review.

*Ginkgo biloba* is a dioecious tree with a history of use in traditional Chinese medicine and has many pharmacologic effects. Ginkgo has vascular protection functions due to its antioxidant effects, free radical scavenging activity, stabilization of membranes, and inhibition of platelet-activating factor. *Ginkgo biloba* extract (GBE), produced from *Ginkgo biloba* leaves, is commonly used in dietary supplements for aliments and has shown excellent clinical effects in many cases. GBE contains terpenoids, flavonoids, alkylphenols, polyprenols, and organic acids. Terpenoids (including ginkgolides and biobalide) and flavonoids are the two major groups of active substances in Ginkgo leaves. The basic structures of ginkgolides, biobalides, and *Ginkgo biloba* flavonol aglycones are shown in Figures 3(a), 3(b), 3(c).

**Figure 4:**
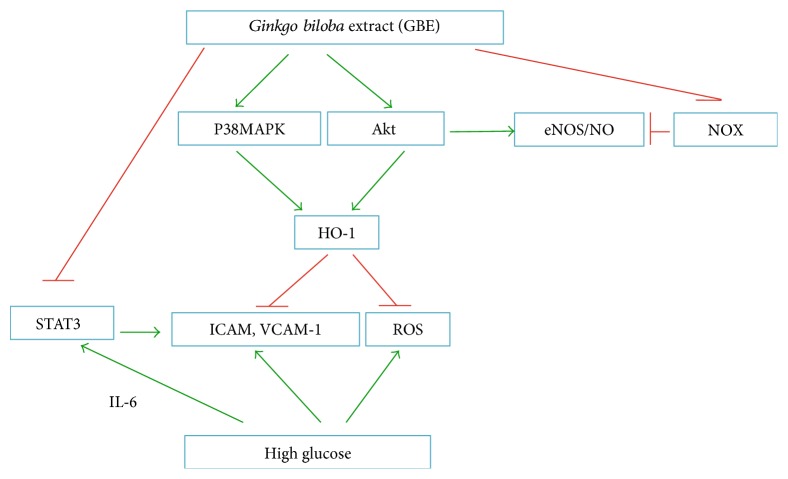
Mechanism for GBE antiatherosclerosis under diabetic conditions. STAT: signal transducer and activator of transcription; ICAM: intercellular cell adhesion molecule-1; VCAM-1: vascular cell adhesion molecule 1.

There have been several reports showing that EGB761, a standard GBE, improves glucose homeostasis, possibly because of increased plasma insulin levels, via protection of pancreatic *β*-cells and/or stimulation of insulin secretion. Cheng et al. reported that GBE (100, 200, and 300 mg/kg) administered orally once a day for 30 days caused a significant dose- and time-dependent reduction in blood glucose levels in diabetic rats. In their study, GBE increased the activities of SOD, CAT, and glutathione peroxidase (GSH-Px) in diabetic rats and resulted in protection of pancreatic *β*-cells [94]. In addition, several reports have shown that GBE lowers blood glucose by improving insulin resistance [95–97]. Thus, GBE may attenuate atherosclerosis in the context of diabetes. According to a study by Lim et al. [98], neointimal formation in balloon-injured carotid arteries is significantly reduced when insulin-resistant rats are treated with EGb761 (100 or 200 mg/kg/day) for 6 weeks, resulting in reduced proliferation and migration of VSMCs. EGb761 (50–200 *μ*g/mL) decreases the proliferation of rat aortic SMCs in a concentration-dependent manner in vitro. In addition, EGb761 at both 100 and 200 *μ*g/mL suppresses the expression of ICAM and VCAM in HUVECs. Zhao et al. [99] found that GBE improves SOD activity and reduces the rate of apoptosis of EPCs within the peripheral blood of diabetic patients in a dose-dependent manner. According to Tsai et al. [100], GBE inhibits high glucose-induced ROS generation, adhesion molecule expression, and monocyte adhesiveness in human aortic endothelial cells (HAECs) via the Akt/eNOS and p38 MAPK pathways. Another study showed that Ginkgolide A at 10, 15, and 20 *μ*M inhibits high glucose-induced IL-4, IL-6, and IL-3 expression in HUVECs. Ginkgolide A attenuates vascular inflammation by regulating the STAT3-mediated pathway [61]. According to a study by Wang et al. [101], treatment with the rutin (30 and 100 *μ*M) significantly restores NO production by decreasing NOX4 mRNA and protein levels and reducing the generation of ROS in HUVECs under high-glucose conditions. Furthermore, rutin at doses of 35 and 70 mg/kg improves endothelium function by restoring impaired NO generation from glucose-triggered endothelial cells and ameliorating the endothelial contraction and relaxation response in thoracic aortas of rats with a high-glucose diet. The potential mechanism for GBE in the treatment of diabetic atherosclerosis is shown in Figure 4.

### 4.2. Tetramethylpyrazine (TMP)

TMP is a biologically active compound isolated from rhizomes of *Ligusticum chuanxiong*, a traditional Chinese medicine (Figure 3(d)). Several studies have shown that TMP exerts antiatherosclerosis effects through promotion of endothelial protection, inhibition of VSMC proliferation, reduction of oxidative stress, and suppression of inflammation and apoptosis. The link between TMP and NO generation has been verified by several researchers. For example, Lv et al. [102] demonstrated that TMP pretreatment in vivo enhances Akt and eNOS phosphorylation. Additionally, Xu et al. reported that Qiong Huo Yi Hao (QHYH), which consists of several herbals based on the “clearing heat and detoxifying” principle of traditional Chinese Medicine, is a potent antioxidant acting to scavenge superoxide anions in endothelial cells treated with high concentrations of glucose [103]. TMP, an active compound in QHYH, has been shown to be the strongest component of QHYH in the prevention of ROS production, functioning to block Akt/eNOS phosphorylation and reduce NO generation in endothelial cells treated with high concentrations of glucose [104]. Xu et al. further demonstrated that TMP ameliorates high glucose-induced endothelial dysfunction by increasing mitochondrial biogenesis through reversing high glucose-induced suppression of SIRTI1 [105]. These findings provide evidence for the endothelial protection function of TMP in the context of hyperglycemia. Studies have shown that TMP can suppress the proliferation of VSMCs [106], and the ERK and p38MAPK pathways may be involved in this process [107]. Additionally, TMP can block LPS-induced IL-8 overexpression in HUVECs at both protein and mRNA levels, which could be attributed to inhibition of the ERK and p38MAPK pathways and the inactivation of NF-*κ*B [108]. The antiapoptotic function of TMP can be attributed to the inhibition of JAK/STAT signal transduction [109].

**Figure 5:**
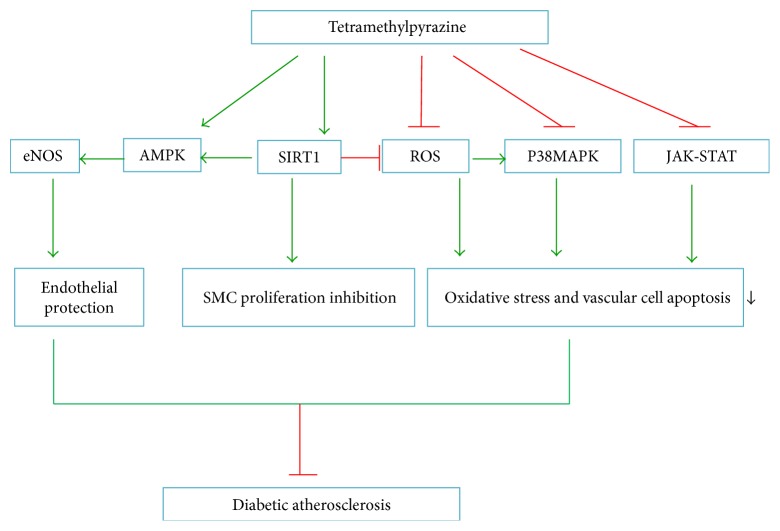
Mechanism through which tetramethylpyrazine protects against diabetic atherosclerosis.

Importantly, Lee et al. [110] investigated the effects of TMP on lipid peroxidation in STZ-induced diabetic mice. The results showed that TMP dose dependently inhibited glucose concentrations, blood urea nitrogen elevation, and the degree of lipoperoxidation. Thus, TMP may be an effective agent for the treatment of diabetes and related vascular complication. The mechanisms through which TMP protects against diabetes are shown in Figure 5.

### 4.3. Danggui

Danggui-Buxue-Tang (DBT) is a well-known traditional formula. Zhang et al. [111] found that oral administration of DBT (3 or 6 g/kg/day for 4 weeks) decreased the concentrations of c-reactive protein and tumour necrosis factor-*α* and resulted in higher survival rates and lower body weight loss in diabetic GK rats; the diabetic atherosclerosis rats were induced by NO inhibition (I-NAME in drinking water, 1 mg/mL) plus a high-fat diet. They also investigated the effects of DBT on blood lipids and the expression of genes related to foam cell formation during the early stage of atherosclerosis in diabetic GK rats. The results demonstrated that DBT could regulate blood lipids and inhibit the expression of *MCP*, *ICAM-1*, and *CD36* genes in the aorta [112]. Galgeun-dang-gwi-tang (GGDGT), a Korean herbal medicine, has traditionally been prescribed for the treatment of diabetes. In a study by Lee et al. [113], lipid metabolism and insulin resistance were shown to be improved by GGDGT in ApoE^−/−^ mice fed with a Western diet. Immunohistochemical staining showed that GGDGT suppressed ICAM expression, whereas the expression of eNOS and IRS-1 was restored by GGDGT in the thoracic aorta and skeletal muscle. GGDGT attenuates endothelial dysfunction via improvement of the NO-cylic guanosine monophosphate signalling pathway and promotes insulin sensitivity in diabetic atherosclerosis.

### 4.4. *Salvia miltiorrhiza* (Danshen) and Salvianolic Acid

*Salvia miltiorrhiza* (Danshen), a traditional Chinese herbal medicine, is commonly used for the prevention and treatment of cardiovascular disease. Salvianolic acid B is the most abundant water-soluble compound extracted from Danshen (Figure 3(e)). Inhibition of inflammation, improvement of antioxidative effects, regulation of leukocyte endothelial adhesion, and modulation of NO production in endothelial cells are involved in the cardiovascular protection mechanism for Danshen and its bioactive compounds [114, 115]. Danshen extract and purified salvianolic acid B exert anti-inflammatory effects by inhibiting iNOS expression and NO production induced by LPS in RAW267.4 macrophages by inducing Nrf2-mediated HO-1 expression [114, 116]. Lee et al. [117] also demonstrated that salvianolic acid B inhibits platelet-derived growth factor-induced neointimal hyperplasia in arteries through induction of Nrf2-dependent HO-1. In addition, salvianolic acid B increases NO production in the endothelium of isolated mouse aortas via inhibition of arginase activity [114]. According to Raoufi et al., administration of salvianolic acid B at doses of 20 or 40 mg/kg/day (i.p.) for 3 weeks significantly decreases serum glucose and improves oral glucose tolerance test (OGTT) in STZ-induced diabetic rats via attenuation of oxidative stress and apoptosis and augmentation of the antioxidant system [118]. The vascular endothelial protective function of *Salvia miltiorrhiza* and salvianolic acid B under high-glucose conditions has been verified both in vitro and in vivo. According to Qian et al. [119], *Salvia miltiorrhiza* (10 *μ*g/mL) significantly decreases vascular endothelial ROS formation in human microvascular endothelial cells exposed to 30 mM glucose. Ren et al. [120] demonstrated that salvianolic acid B significantly restores eNOS in STZ-induced diabetic rats and decreases the levels of NOX and endothelial cell apoptosis. The mechanism through which salvianolic acid B protects against diabetic atherosclerosis is shown in Figure 2.

### 4.5. Catalpol

Catalpol is the most abundant bioactive component in the roots of *Rehmannia glutinosa* (Figure 3(f)). Catalpol ameliorates plasma glucose in STZ-induced diabetic rats [121], and total cholesterol, triglycerides, and LDL cholesterol are reduced, whereas HDL cholesterol is elevated when the rats fed with high-cholesterol chow are treated with catalpol [122]. Additionally, atherosclerotic lesions and inflammatory markers are markedly reduced in the catalpol group, and catalpol attenuates atherosclerotic lesions and delays the progression of atherosclerosis in alloxan-induced diabetic rabbits. These protective effects are associated with regulation of glucose insulin homeostasis and inhibition of oxidative stress and inflammation [123].

### 4.6. Resveratrol

**Figure 6:**
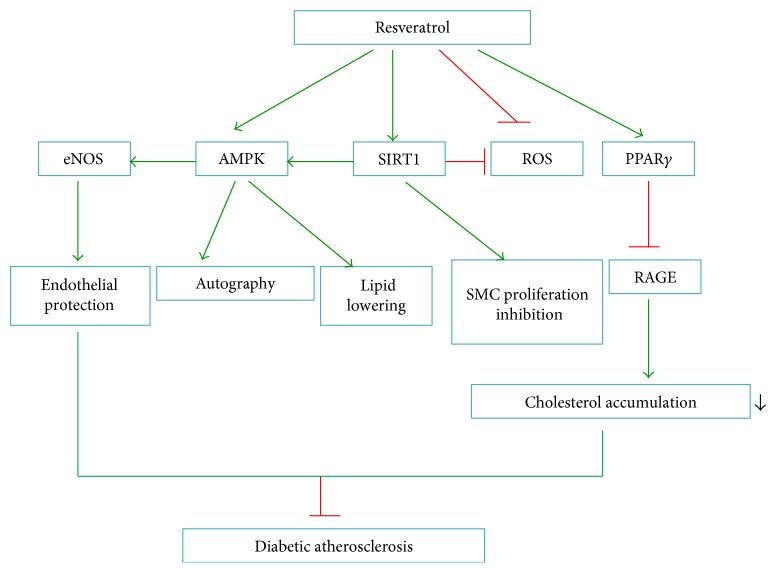
Antiatherosclerotic mechanism of resveratrol under diabetic conditions. RAGE: advanced glycosylation end product receptor.

Resveratrol (*trans*-3,5,4′-trihydroxystilbene) is a natural polyphenol phytoalexin (Figure 3(g)) with various biological effects. The beneficial cardiovascular effects of this drug are attributable to its anti-inflammatory, antioxidative stress, endothelial protection, antiplatelet, and insulin-sensitizing effects [124]. Resveratrol increases NO bioavailability by regulating SIRT1, AMPK, and ROS. According to a study by Yang et al. [125], resveratrol restores the NO bioavailability impaired by high glucose in human endothelial cells in a SIRT1-dependent manner. Other studies have shown that resveratrol downregulates NF-*κ*B induced by high glucose in smooth muscle cells and decreases the proliferation and migration of smooth muscle cells, a function similar to *miR-138* inhibitors, which result in upregulation of SIRT1 [41]. Zhang et al. [126] demonstrated that resveratrol prevents impairment of the effects of AGEs on macrophage lipid homeostasis partially by suppressing RAGEs via PPAR*γ* activation, thus providing new insights into the protective roles of resveratrol against diabetic atherosclerosis. Furthermore, resveratrol lowers lipid levels and decreases hepatic lipid accumulation by stimulation of AMPK dependent on SIRT1 activity. These findings suggest that resveratrol may have potential therapeutic effects through regulation of dyslipidaemia-associated atherosclerosis in diabetes by targeting SIRTI/AMPK signalling [127, 128]. The underlying antiatherosclerotic mechanisms of resveratrol in the context of diabetes are illustrated in Figure 6.

### 4.7. Curcumin

Curcumin (diferuloylmethane) is a constituent of turmeric (*Curcuma longa*) spice. Decreased serum LDL levels and increased serum HDL levels were observed after patients with atherosclerosis were administered 10 mg curcumin twice daily for 28 days [129]. Usharani et al. [130] showed that administration of a standardized preparation of curcuminoids (NCB-02, two capsules containing 150 mg curcumin, twice daily) for 8 weeks significantly improved the endothelial function of patients with type 2 diabetes mellitus. Curcumin also blocks oxidative stress and inflammation by modulating PPAR*γ* and Nrf2 activity [131]. Zheng et al. showed that the curcumin analogue L3 alleviates dyslipidaemia and hyperglycemia and reduces oxidative stress in diabetic mice induced by STZ and a high-fat diet. Additionally, L3 effectively decreases lectin-like oxidized low-density lipoprotein receptor-1 expression in the aortic arch. These results suggested that curcumin ameliorates diabetic atherosclerosis through multiple mechanisms [132].

## 5. Future Perspectives

Insulin resistance and hyperglycemia are associated with diabetic atherosclerosis, and endothelial dysfunction, vascular inflammation, myeloid cell recruitment, oxidative stress, VSMC phenotype changes, and platelet hyperreactivity all contribute to diabetic atherosclerosis. As reported recently, crosstalk between macrophage polarization and autophagy may be involved in diabetes and related atherosclerosis complications [133, 134]. Extensive preclinical studies have identified the molecule targets and herbs that act on these targets as potential therapeutic agents for the management of diabetic atherosclerosis (see Table 2). However, currently, most clinical studies have small sample sizes and are not performed using a randomised design. The lack of high-quality clinical trials hampers the application of herbal medicines in patients with diabetic atherosclerosis. Therefore, more rigorous clinical trials of herbs on diabetic atherosclerosis, with large sample sizes and a randomised, controlled design, are needed. Furthermore, detection of new molecules and signalling cascades that regulate diabetes and atherosclerosis will help to improve treatment approaches owing to the multifaceted characteristics of diabetic atherosclerosis. Investigation of the mechanisms of multitargeted effects of herbs will also help to establish novel drugs for the treatment of diabetes and diabetic atherosclerosis. In the future, the combination of herb with western medicine may also facilitate the treatment of diabetic atherosclerosis. Thus, further studies on drug interactions and safety are needed.

## Figures and Tables

**Table 1:** MicroRNA involved in diabetic atherosclerosis.

miR type	Animal model	Mechanism	Target gene	Reference
miR33	STZ-induced diabetic Ldlr−/− mice	Increased macrophages and lipid content	ABCA1	[37]
miR 125b	Type 2 diabetic db/db mice	Increased inflammatory gene expression	SUV39H1	[38]
miR 200	Type 2 diabetic db/db mice	Increased inflammatory gene expression	Zeb1	[39]
miR 504	Type 2 diabetic db/db mice	Vascular smooth muscle phenotype change	Grb10	[40]
miR138	Type 2 diabetic db/db mice	Vascular smooth muscle phenotype change	SIRT1	[41]

ABCA1: ATP-binding cassette transporter A1; SIRT1: silent information regulator 1.

**Table 2:** Fundamental application of herb in diabetic atherosclerosis.

Drug	Dosage	Administration	Model	Reference
In vitro studies				
GBE	100 *μ*g/mL	Incubation 18 h	HAECs cultured in high glucose	[100]
Ginkgolide A	10, 15, 20 *μ*M	Preincubation 30 min	HUVECs cultured in high glucose	[61]
Rutin	30, 100 *μ*M	Preincubation 30 min	HUVECs cultured in high glucose	[101]
TMP	10 *μ*M	Incubation 48 h	bEnd.3 and HUVECs cultured in high glucose	[104]
TMP	30 *μ*mol/L	Incubation 48 h	bEnd.3 and HUVECs cultured in high glucose	[105]
*Salvia miltiorrhiza*	10 *μ*g/mL	Incubation 48 h	HMECs cultured in high glucose	[119]
Resveratrol	1 *μ*mol/L	Preincubation 24 h	HUVECs cultured in high glucose	[70, 125]
In vivo studies				
EGb761	100, 200 mg/kg/d	p.o. 6 weeks	Obesity and insulin-resistant rats	[98]
Rutin	35, 70 mg/kg/d	p.o. 12 weeks	SD rats fed with high glucose	[101]
TMP	10, 25, 50 mg/kg	i.p. 2 weeks	STZ-induced diabetic mice	[110]
DBT	3, 6 g/kg/d	p.o. 4 weeks	Nitric oxide inhibition plus high-fat diet-fed rats	[111]
DBT	3, 6 g/kg/d	p.o. 4 weeks	Nitric oxide inhibition plus high-fat diet rats	[112]
GGDGT	200 mg/kg/d	p.o. 12 weeks	Western diet-fed ApoE−/− mice	[113]
Salvianolic acid B	80, 160 mg/kg/d	p.o. 6 weeks	STZ-induced diabetic atherosclerosis	[120]
Catalpol	5 mg/kg/d	p.o. 12 weeks	Hyperlipidemic diet plus alloxan diabetic-induced rabbit	[123]

HUVECs: human umbilical vein endothelial cells; HACEs: human aortic endothelial cells; bEnd.3: murine brain microvascular cell line; HMECs: microvascular endothelial cells; DBT: Danggui-Buxue-Tang; GGDGT: Galgeun-dang-gwi-tang; STZ: streptozotocin.
